# A Protocol of a Guideline to Establish the Evidence Ecosystem of Acupuncture

**DOI:** 10.3389/fmed.2021.711197

**Published:** 2022-02-15

**Authors:** Qin Wang, Nian Li, Juan Li, Ying He, Yuxi Li, Dongling Zhong, Xiaobo Liu, Jin Fan, Rongjiang Jin, Deying Kang, Yonggang Zhang

**Affiliations:** ^1^Department of Evidence Based Medicine and Clinical Epidemiology, West China Hospital, Sichuan University, Chengdu, China; ^2^Department of Medical Affairs, West China Hospital, Sichuan University, Chengdu, China; ^3^School of Health Preservation and Rehabilitation, Chengdu University of Traditional Chinese Medicine, Chengdu, China; ^4^Department of Periodical Press and National Clinical Research Center for Geriatrics, Nursing Key Laboratory of Sichuan Province, West China Hospital, Sichuan University, Chengdu, China

**Keywords:** acupuncture, guideline, evidence ecosystem, living systematic reviews, clinical trial

## Abstract

This is a protocol for developing a guideline to establish the evidence ecosystem of acupuncture. It describes all steps that will be followed in line with the World Health Organization Handbook for Guideline Development and the Reporting Items for practice Guidelines in Healthcare (RIGHT). The key steps included guideline protocol development, guideline registration, systematic review of acupuncture evidence issues, systematic review of methods for establishing evidence ecosystem, survey of acupuncture stakeholders regarding potential acupuncture evidence issues, development of potential items for guidelines, Delphi method for guideline item development, consensus meeting, drafting guideline, peer review, approval, and publishing. This future guideline will help to establish evidence ecosystem of acupuncture, which will facilitate the application of acupuncture in clinical practice.

## Introduction

The United Nations Educational, Scientific, and Cultural Organization (UNESCO) had placed acupuncture on the Representative List of the Intangible Cultural Heritage of Humanity (1) and this therapy had been deemed safe and effective. Acupuncture is an important Chinese medicine treatment method suitable for a wide spectrum of diseases (2). More than 60,000 randomized controlled trials (RCTs), 6,000 systematic reviews, and 1,000 recommendations had been published, while some studies were published in top journals (3, 4), which promoted the use of acupuncture worldwide.

Acupuncture clinical practice should be based on high-quality evidence, which could help in decision-making. Thus, acupuncture research should provide sufficient data to enable funders, reviewers, and steering committees to appraise the scientific and methodological rigor of the studies, and for the researchers to replicate and implement these studies (5). However, many RCTs were of low quality and were characterized by incorrect random allocation, vague statistical analyses, and methodological issues (6). Meanwhile, effect sizes do not provide policymakers with information on how an intervention might be replicated in their specific context, or whether trial outcomes will be reproduced. In addition, some systematic reviews were also of low quality, containing insufficient comparisons and out-of-date, thereby resulting in redundancy and research gaps (7). Furthermore, some acupuncture guidelines contained insufficient interpretations and explanations, and the majority was in the Chinese language (8, 9), thereby decreasing the promotion of acupuncture. High-quality and up-to-date systematic reviews require substantial time and resources, and although the synthesis of evidence is directly affected by the quality of primary research (10), there has been little effort to connect evidence generation to evidence synthesis. These issues had been highlighted and exacerbated with an increasing use of acupuncture. Relevant, accessible, up-to-date, and trustworthy syntheses of high-quality evidence are urgently needed in clinical practice. Although thousands of RCTs had been conducted, the authors often rushed to publish or to communicate through non-peer-reviewed preprints. With the development of acupuncture, it is also worthy to pay attention to the component issue of acupuncture (11). Meanwhile, process evaluation within trials, which has been used to assess fidelity and quality of implementation, clarify causal mechanisms, and identify contextual factors associated with variation in outcomes should also be paid attention to. Still, there were limited studies about the process evaluation within acupuncture trials. A new model was needed to address these challenges and to help connect evidence generation, synthesis, and decision-making. Therefore, the development of an evidence ecosystem is a possible solution.

**Figure 1:**
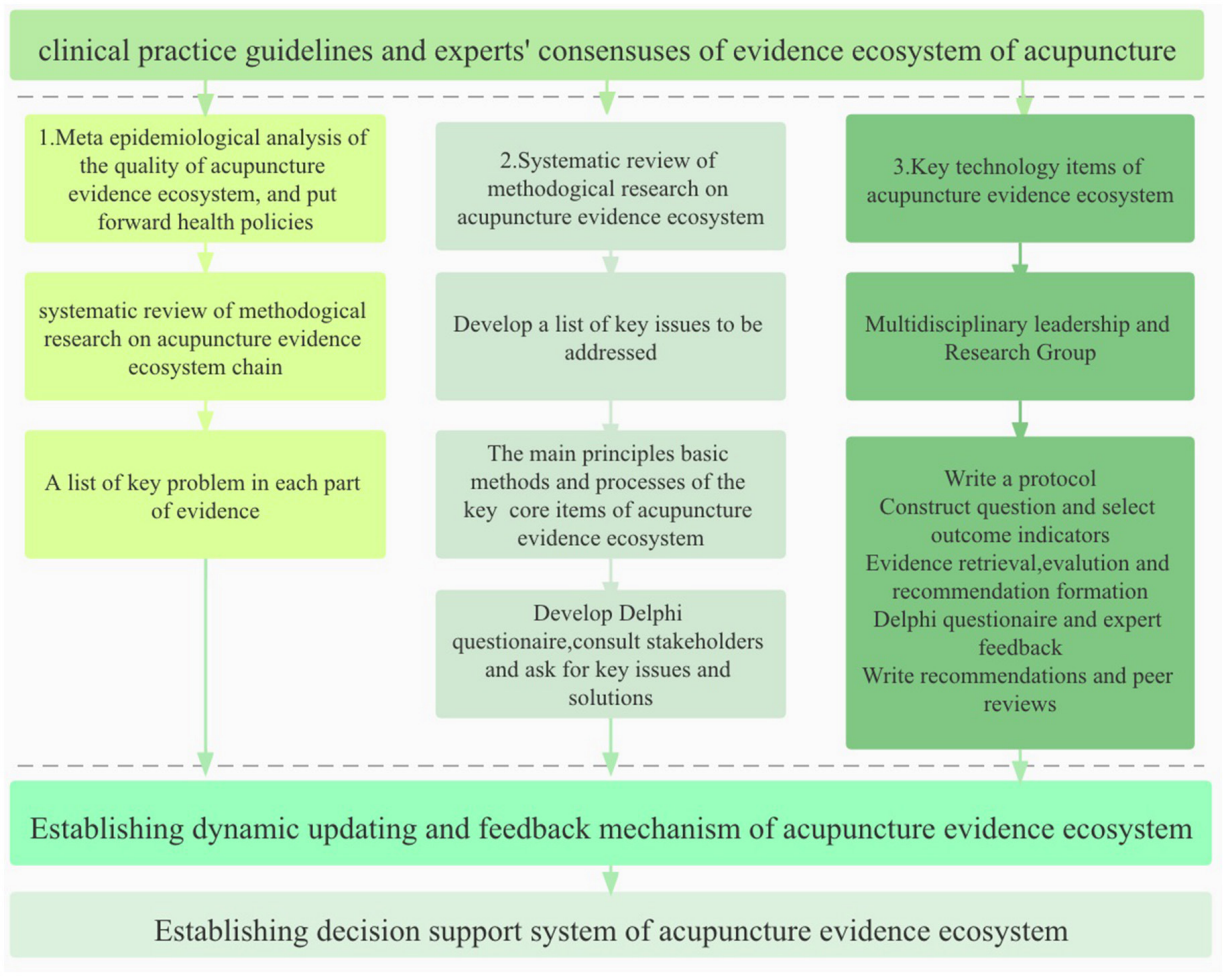
The process of establishing an acupuncture evidence ecosystem.

The evidence ecosystem (7, 10, 12–14) was a concept developed by the MAking GRADE the Irresistible Choice (MAGIC) foundation (14) to promote the continuous cycle of evidence production, evidence synthesis, and guidance generation. Up-to-date evidence is needed to flow efficiently between different stakeholders. These stakeholders included evidence producers (conducting primary research), evidence synthesizers (summarizing the research into systematic reviews or evidence syntheses), evidence processors and disseminators (producing evidence-informed decision products such as health systems guidance and policy briefs), and evidence implementers [individuals responsible for implementing evidence-informed decisions within health systems (7, 10, 15–18), such as program managers and policymakers, and those involved in delivering and using health services, such as service providers and citizens]. In addition, there is no acupuncture evidence ecosystem, so it is urgent to establish it. Establishing an acupuncture evidence ecosystem (14) involved different stakeholders, such as researchers, funders, and reviewers in literature reviews, process evaluation, workshops, and discussions at conferences and seminars. Thus, a guideline should be developed. The guideline should not only include treatment methods of acupuncture, but also realized the integration and utilization of acupuncture evidence-based decision-making information resources from different sources. To ensure a high-quality development, a protocol is needed (Figure 1). The current study was the protocol which introduced the key steps for the development of the guideline. It would increase transparency and objectivity of the guideline.

## Methods

### Guidance of Guideline

The guideline will be evidence-based. It will be developed according to the World Health Organization Handbook for Guideline Development and reported according to the Reporting Items for practice Guidelines in Healthcare (RIGHT). It has been registered on the Global Practice Guidelines Registry Platform (registration number: IPGRP-2021CN106).

### Guideline Group

The guideline project was launched by West China Hospital of Sichuan University in April 2021. Chinese Evidence-based Medicine Center or Cochrane China would provide technical support. The guideline working groups will be established in mid-2021 and will comprise four groups: a guideline steering committee, a guideline formulation group, a guideline secretary group, and an external review group.

The steering committee group will comprise a clinical chairman, a methodology chairman, an executive chairman, and acupuncture experts. Their responsibilities will be as follows: determine the scope according to PICO strategy; establish a guideline secretary group, evidence evaluation group, and consensus expert group; manage the statement of conflict of interest; supervise the process of guideline formulation; approve the recommendations; and monitor and evaluate the revised guideline.

Experts of evidence-based medicine, acupuncturists, editors, policymakers, and computer scientists will be invited as the guideline formulation group. Their responsibilities will be as follows: perform a rapid and high-quality retrieval of the relevant evidence, evaluation, synthesis, and quality classification; generate a summary table and draft recommendations; draft, modify, and improve the guideline; and promote and disseminate the guideline.

Students majoring in evidence-based medicine and acupuncture will be selected as the guideline secretary group. Their responsibilities will be as follows: coordinate and organize the work during guideline formulation; investigate the essential clinical problems and outcomes; organize three Delphi expert consensus conferences (online or face-to-face); draft, modify, and improve the guideline; and conduct data analysis and evidence synthesis.

The external review group will consist of acupuncture stakeholders, including evidence-based medicine experts, acupuncture experts, TCM experts, editors, policymakers, computer scientists, and patients. Their responsibilities will be as follows: evaluate guideline scope and the issues covered and review the draft guideline.

We will use a systematic approach to design the whole evidence ecosystem of acupuncture, including establishing clear descriptions of ecosystem theory, processing evaluations in acupuncture clinical trials, and identifying of key process questions in ecosystem. In addition, each process in the ecosystem will be different, and the guidance facilitates planning and conducting a process ecosystem.

All members involved in the guideline development will declare their interests and relevant relationships, and also complete a statement of interest form. If conflicts of interest exist, the expert will be excluded from participation in evidence evaluation and recommendation.

### Problems With Guideline Construction

It is important to develop a high-quality and useful acupuncture evidence ecosystem. We will define the problems in guideline construction using the Population, Intervention, Comparator, Outcome strategy (PICO).

### Literature Search

We will search RCTs, systematic reviews, meta-analyses, overviews of systematic reviews and clinical practice guidelines, clinical decision analyses, health technology evaluations, and health policy research methods using the following electronic databases: PubMed, EMbase, CBM, CNKI, VIP, and WanFang Data. We will search the gray literature using the following websites to collect potential guidelines: the National Guideline Clearinghouse (Agency for Healthcare Research and Quality), Canadian Medical Association InfoBase, Guidelines International Network, PEDro, Trip Database, American College of Physicians Clinical Recommendations, Australian Government, National Health and Medical Research Council, Health Services/Technology Assessment Texts, Institute for Clinical Systems Improvement, National Institute for Health and Clinical Excellence (NICE) Guidance, NICE Pathways, New Zealand Guidelines Group, Scottish Intercollegiate Guidelines Network (SIGN), and the WHO guidelines approved by the Guidelines Review Committee. Search terms are shown in Table 1.

**Table 1:** Search strategy used for PubMed database.

**No**.	**Search items**
1	Randomized controlled trial ti,ab
2	Controlled clinical trial ti,ab
3	Randomized ti,ab
4	Randomly ti,ab
5	Or 1–4
6	Acupuncture therapy mesh
7	Acupuncture therapy ti,ab
8	Acupuncture treatment ti,ab
9	Pharmacoacupuncture treatment ti,ab
10	Acupuncture ti,ab
11	Acupoints ti,ab
12	Acupunct ti,ab
13	Manual acupuncture ti,ab
14	Body acupuncture ti,ab
15	Scalp acupuncture ti,ab
16	Auricular acupuncture ti,ab
17	Auriculotherapies ti,ab
18	Electroacupuncture ti,ab
19	Fire needling ti,ab
20	Warm needling ti,ab
21	Elongated needle ti,ab
22	Intradermal needling ti,ab
23	Dermal needle ti,ab
24	Plum blossom needle ti,ab
25	Or 6–24
26	5 and 25

### Study Inclusion and Exclusion Criteria

The following inclusion criteria will be used: RCTs, systematic reviews, meta-analyses, overviews of systematic reviews and clinical practice guidelines, clinical decision analyses, health technology evaluations, and health policy research methods in acupuncture. The following exclusion criteria will be used: non-Chinese and non-English documents; conference abstracts, reviews, and so on; complete unavailable data; old versions of guidelines, consensus, standards, procedures, and specifications formulated by the same institution; organization or management-related guidelines, standards procedures, and specifications formulated and released by non-associated societies and groups; interpretations, translations, plans, reviews, and other articles related to guidelines, consensuses, standards, procedures, and norms; research on compliance with guidelines, consensuses, standards, procedures, and norms; lastly, guidelines, standards, processes, and normative after-effect evaluation research, etc.

### Study Selection

Two independent reviewers (QW and YH) will screen titles and abstracts to identify potentially eligible documents, which will be retrieved in full text for final review. Duplicate records will be removed. Any disagreements will be discussed and resolved by a third reviewer (YGZ).

### Data Extraction

We will design a data extraction form based on study characteristics and conduct a pre-experiment. The following content will be extracted: author, publication year, published journal, disease, sample size, location, and quality of evidence. If extra information about the study is required, we will contact the author.

### Quality Assessment of Included Studies

We will assess the methodological quality of the included studies using several tools (Table 2). We will assess the quality of evidence using the Grading of Recommendations Assessment, Development, and Evaluation (GRADE) (15–17) system. We will evaluate the methodological quality and reporting quality of the included guidelines using the Appraisal of Guidelines for Research and Evaluation (AGREE) II tool (18–21) and the RIGHT statement (22), respectively. The quality of evidence will be divided into four levels: high (A), medium (B), low (C), and very low (D) basing on GRADE system. We will use internationally agreed standards to make transparent recommendations (Table 3).

**Table 2:** Document types and evaluation tools.

**Steps**	**Research type**	**Methodological quality assessment tools**	**Standard of medical research report**
(1) Evidence production	Randomized controlled trial (RCT)	Cochrane risk of bias assessment tool	CONSORT
	Non-randomized experimental study	MINORS items	TREND
	Cohort study	NOS scale	TREND
	Case-control study	NOS scale	TREND
	Animal experiment	STAIR list	ARRIVE GUIDELINE
	Economic research	Drummond standard	CHEERS
(2) Evidence synthesis	Systematic review/Meta-analysis	AMSTAR 2 tool	PRISMA (RCT)
		OQAQ scale	MOOSE (observational research)
		SQAC scale	
	Overviews of systematic reviews	AMSTAR 2 tool	
		OQAQ scale	
(3) Creating guidelines and conducting health technology assessments	Clinical practice guidelines	AGREE II tool	
	Health technology assessment	Checklist for HTA report	
	Health policy research	Experimental study: Cochrane EPOC evaluation method	
		Observational research: quality evaluation criteria for Hilton's effective public health policy project development	
(4) Dissemination of evidence	Clinical practice guidelines	AGREE II tool	
	Health technology assessment	Checklist for HTA report	
	Decision aids		
(5) Applied evidence	Decision support system		
(6) Assessment and improvement practices	Real-world study		

*CONSORT, consolidated standards of reporting trials; MINORS, methodological index for non-randomized studies; TREND, transparent reporting of evaluations with non-randomized designs; NOS, Newcastle–Ottawa Scale; CHEERS, consolidated health economic evaluation reporting standards; AMSTAR 2, assessment of multiple systematic reviews measurement tool; OQAQ, Oxman–Guyatt Overview Quality Assessment Questionnaire; SQAC, Sack's Quality Assessment Checklist; MOOSE, Meta-analyses Of Observational Studies in Epidemiology; HTA, Health Technology Assessment; AGREE II, Appraisal of Guidelines for REsearch and Evaluation II; Cochrane EPOC evaluation method, Cochrane Effective Practice and Organization of Care review group; ARRIVE, animal research, reporting of in vivo experiments; PRISMA, preferred reporting items for systematic reviews and meta-analyses*.

**Table 3:** Factors that determine the direction and strength of a recommendation.

**Factor**	**How the factor influences the direction and strength of a recommendation**
Quality of the evidence	The quality of the evidence across outcomes is critical to decision-making and informs the strength of the recommendation. Higher quality evidence is associated with a greater likelihood of a strong recommendation.
Values and preferences	This describes the relative importance assigned to health outcomes by those affected by them; how such importance varies within and across populations; and whether this importance or variability is surrounded by uncertainty. The less uncertainty or variability there is about the values and preferences of people experiencing the critical or important outcomes, the greater the likelihood of a strong recommendation.
Balance of benefits and harms	This requires an evaluation of the absolute effects of both benefits and harms (or downsides) of the intervention and their importance. The greater the net benefit or net harm associated with an intervention or exposure, the greater the likelihood of a strong recommendation in favor or against the intervention.
Resource implications	This pertains to how resource-intense an intervention is, whether it is cost-effective, and whether it offers any incremental benefit. The more advantageous or clearly disadvantageous the resource implications are, the greater the likelihood of a strong recommendation either for or against the intervention.
Priority of the problem	The problem's priority is determined by its importance and frequency (i.e., burden of disease, disease prevalence, or baseline risk). The importance of the problem increases in tandem with the likelihood of a strong recommendation.
Equity and human rights	The greater the likelihood that the intervention will reduce inequities, improve equity, or contribute to the realization of one or several human rights as defined under the international legal framework, the greater the likelihood of a strong recommendation.
Acceptability	Greater acceptability of an option to all or most stakeholders is associated with greater likelihood of a strong recommendation.
Feasibility	Greater feasibility of an option from the standpoint of all or most stakeholders is associated with greater likelihood of a strong recommendation. Feasibility overlaps with values and preferences, resource considerations, existing infrastructures, equity, cultural norms, legal frameworks, and many other considerations.

### Data Synthesis and Presentation

A synthesis of qualitative research will be conducted as part of the scope of the guideline and this will also help to define and to refine the questions. A mixed-methods synthesis will combine a meta-analysis of quantitative data and a qualitative analysis. We will summarize the recommendations for the acupuncture evidence ecosystem. The main outcomes will be as follows: author, title and subtitle, type and version, implementation status, target disease, population, user, country or region, literature search method, funding, and patient preferences. The outcome of qualitative research is patient preferences. We will evaluate the effects by using “Preferences about Therapy Questionnaire” on a 5-point Likert scale. We will perform a systematic review of the main principles, basic methods, and procedures for formulating key core technical items of the evidence ecosystem.

### Development of Recommendations

Once the evidence is retrieved, synthesized, and assessed, the evidence will be used to develop recommendations. The recommendations will be surveyed by 3 rounds of Delphi methods. We will conduct an online-based Delphi process. It is an accepted method in medical research to gain consenting information from a group of experts. The anonymous multi-step survey comprised three rounds, starting with open-ended questions and ending with a consensus-scoring assessment of the guideline. Design and analysis methods will be adopted and modified from a recent guideline for the World Health Organization Handbook for Guideline Development. The online Delphi process is conducted using the survey tool Questionnaire star. We will submit them to the guideline-steering committee for recommendations. Then, the draft of the guideline will be developed. A full draft will recirculate for further review before being revised and approved.

### Peer Review

The guideline will undergo peer review. The review and response processes will be recorded. The guideline will be revised after discussion with the peer review comments. Peer review opinions will be submitted to the guideline steering committee for further assessment. The next version of the guideline will be developed.

### Dissemination of Guidelines

Dissemination involves making guidelines accessible, advertising their availability, and distributing them widely. The guidelines will be disseminated through association. The guideline will be submitted to peer review journals, such as the Journal of Evidence-based Medicine or other journals.

### Establishment of the Evidence Ecosystem on Acupuncture

The guideline is used for establishing the evidence ecosystem of acupuncture. After the approval of the guideline, the evidence ecosystem will be established.

## Discussion

This is a protocol for the development of a guideline, which will be used to establish an acupuncture evidence ecosystem. Several challenges of developing the guideline should be considered. First, sustainability is a potential issue in generating the data. Thus, an appropriate strategy should be considered when developing the guideline. Seeking assistance from the central government or industry may be useful in developing the ecosystem. A potentially good example from the Cochrane Collaboration may be helpful. We should move to a long-term, inexpensive, and sustainable structure with a website that is more accessible and useful to end-users. Collaboration with associations or researchers would be useful. In addition, the ecosystem will be used to strengthen clinical practice, which we will provide a guidance for carrying out the process evaluation of acupuncture clinical trials. Therefore, efficient flow between evidence producers, evidence synthesizers, evidence processers and disseminators, and evidence implementers in the evidence ecosystem should be well defined. International scientists should also be invited to contribute to the ecosystem and assist with decision-making. Third, because the quality of evidence ecosystem of acupuncture is still uncertain, the recommendations should be carefully considered (23, 24). Fourth, the ecosystem should be considered a living system. Thus, the development of a living guideline should be considered, which will benefit the development of the evidence ecosystem. Fifth, the ecosystem should further consider the development of acupuncture discipline, such as acupuncture manipulation metrology which has occupied an important strategic position (25).

The acupuncture evidence ecosystem model will illustrate how evidence is transferred between different key stages to strengthen health systems and inform care. It will show the importance of “closing the loop” between evidence producers, synthesizers, disseminators, and users. It will also help to process evaluation and to provide potential progress in developing high-quality acupuncture trials. Although the acupuncture evidence ecosystem regarding evidence generation, synthesis, and translation contains thriving areas, others remained unclear. Recognition of these strengths and weaknesses, and the identification of future paths to a more robust system would allow health care and public institutions to focus on the real problems with acupuncture evidence rather than the purported problems highlighted by universal critics. A future priority is to further strengthen initiatives to establish an acupuncture evidence ecosystem, to translate evidence into policy and practice, and to sustain the capacity for continuous technical support for health system policy development and implementation.

In summary, this protocol provides a plan for developing a guideline. The future guideline will help to establish evidence ecosystem of acupuncture, which will facilitate the application of acupuncture in clinical practice.

## Author Contributions

YZ and DK: design the study. NL, YH, DZ, XL, JF, and RJ: data collection and analysis. QW and YZ: draft the protocol. YZ and JL: revise the protocol. All authors contributed to the article and approved the submitted version.

## Funding

This protocol was supported by the National Natural Science Foundation of China (Grant No. 82004213) and the Project of Sichuan Provincial Department of Science and Technology (Grant No. 2021YFH0191).

## Conflict of Interest

The authors declare that the research was conducted in the absence of any commercial or financial relationships that could be construed as a potential conflict of interest.

## Publisher's Note

All claims expressed in this article are solely those of the authors and do not necessarily represent those of their affiliated organizations, or those of the publisher, the editors and the reviewers. Any product that may be evaluated in this article, or claim that may be made by its manufacturer, is not guaranteed or endorsed by the publisher.
